# Piriformis syndrome: a systematic review of case reports

**DOI:** 10.1186/s12893-025-03202-2

**Published:** 2025-10-09

**Authors:** Giovanni Monteleone, Giorgio Stevanato, Maurizio Alimandi, Edoardo Cappa, Roberto Sorge

**Affiliations:** 1 Department of Biomedicine and Prevention, University of Rome Tor Vergata, Rome, Italy; 2 Neurosurgery Unit, Dell’Angelo Hospital, Mestre (Ve), Italy; 3 Department of Clinical and Molecular Medicine, Sapienza University of Rome, Rome, Italy; 4 Department of Experimental Medicine, University of Rome Tor Vergata, Rome, Italy; 5 Department of Systems Medicine, University of Rome Tor Vergata, Rome, Italy

**Keywords:** Case reports, Intrapelvic sciatic nerve entrapment, Piriformis muscle sciatica, Piriformis syndrome

## Abstract

**Background:**

To study the medical history, diagnosis, management, and treatment results of piriformis syndrome (PS).

**Methods:**

Articles published between 1980 and 2024 reporting cases of PS or piriformis muscle sciatica (PMs) case/case series were included. We excluded articles that did not report anagraphic data for singular cases, diagnostic procedure, therapy, and outcome for each case. We searched PubMed database and we retrieved articles from references.

We used the Preferred Reporting Items of Systematic Reviews (PRISMA) guidelines to conduct a systematic review of the literature to identify all published cases of PS or piriformis muscle sciatica (PM). Data for all cases were collected in a database and analysed using statistical software (Statistical Package for the Social Sciences for Windows).

**Results:**

Of the 235 articles screened, 97 were included. Data from 212 patients (117 females and 95 males, mean age 43.6 ± 14.8) were collected. 38.2% of the patients in this study had a history of blunt / indirect pelvic trauma or piriform muscle (PM) stress due to vigorous physical activity/sport. 9.0% (19/212) of the patients had previously failed lumbar spine surgery.

Before treatment, the diagnosis of PS/PMs was corroborated in 29.7% of patients by intrapelvic magnetic resonance imaging (MRI); 50.5% of the patients had a PS clinical diagnosis.

Conservative treatments were effective in treating PS/PMs in 41.1% of patients; 58.9% of patients required surgical treatments. In the group of patients with PS diagnosis made without instrumental finding, the OR of surgical treatment failure occurrence was 5.3.

After treatment, the most frequent causes of PS/PMs identified by intraoperative or instrumental findings were the anatomical variant of PM or SN (12.7%) followed by pyomyositis (9.4%) and PM hypertrophy (7.5%). 47.6% of the patients had no instrumental or intraoperative findings.

**Conclusions:**

Intrapelvic MRI was the instrumental examination most frequently used to confirm the diagnosis of PS/PMs prior to treatment.

The PS causes most frequently identified were the anatomical variant of PM or SN. In the group of patients with PS diagnosis made without instrumental finding, the OR of surgical treatment failure occurrence was 5.3.

To reduce the number of cases of persistent pain after treatment for suspected PS, it is advisable to support the clinical diagnosis through all available instrumental diagnostic procedures. However, considering all the risks that SN surgery can cause, all nonsurgical treatments should be encouraged prior to surgery.

**Trial registration:**

PROSPERO Reg. No. CRD42025641061.

**Supplementary Information:**

The online version contains supplementary material available at 10.1186/s12893-025-03202-2.

## Introduction

Sciatica is a common symptom with a lifetime incidence ranging from 13 to 40% in the entire population; the equivalent annual incidence of sciatica episodes ranges from 1 to 5% [1].

Neuroradiological studies affirm that 85% of sciatica cases are associated with intervertebral disc disease [2]. Pathological conditions that develop in the pelvis and cause entrapment or impingement with the SN can simulate sciatica. In the pelvis, there are close spatial relationships between the SN and the piriformis muscle (PM). The growth of expansive masses inside or intrinsic alterations in PM can trigger symptoms of low back/buttock pain and leg pain, simulating vertebral sciatica. Robinson called this painful condition Piriformis syndrome in 1947, which means sciatica due to an abnormal PM [3].

PS was first added in 2019 to the 11th revision of the International Statistical Classification of Diseases and Related Health (ICD-11). Its prevalence is estimated to range between 0.3% and 6% of all cases of low back pain and/or sciatica, with an incidence in the United States of approximately 2.4 million per year [4, 5].

Instrumental exams often do not have findings that explain the pathogenesis of sciatica, and the diagnosis of PS is often based only on clinical features, excluding vertebral causes. Hopayian and Danielyan [6], in a systematic review, identified four common symptoms to consider in the diagnosis of PS: buttock pain, pain aggravated while sitting, external tenderness near the greater sciatic notch, and pain in any manoeuvre that increases PM tension and limits straight leg raising.

The difficulty in identifying the cause of PS hampers the right therapeutic choice and can lead to failure of nonsurgical or surgical treatment.

PS affects not only athletes, but also the working-age population, which means that from a social point of view, many professionals, including physiatrists, sports medicine physicians, orthopaedic surgeons, neurosurgeons, and anesthesiologists, are involved in its clinical management, from diagnosis to treatment.

Although the literature includes many case reports and series of PS cases, there is no common approach to instrumental diagnosis or treatment, and a clinical evaluation compatible with PS is often only confirmed ex adiuvantibus [7, 8].

As in medical records, case reports and small case series collect diagnostic and anamnestic details of patients, which are usually excluded in large series studies.

Case reports are written retrospectively with many details that allow the events of a disorder to be reconstructed [9].

In the challenging clinical scenario of PS, a systematic review of case reports provides a detailed picture of the diagnostic and therapeutic criteria applied in specific conditions.

The current systematic review provides an evidence-based assessment of the medical history, instrumental diagnostic approach, treatment, and outcome of published cases.

## Methods

This systematic review was conducted in accordance with PRISMA and was registered in PROSPERO (CRD42025641061).

### Search strategy

We performed a search on PubMed, with the following keywords: (Piriformis, syndrome) AND case report, (Piriformis, syndrome) AND series; (Pyriformis, syndrome) AND case report; (Pyriformis, syndrome) AND series; (Piriformis, sciatica) AND case report; (pyramidal, sciatic, syndrome) AND case report. The references of retrieved articles were searched for further articles. The search was carried out by 2 investigators.

### Evaluation of risk of bias (quality)

Both authors separately evaluated the calibre of the included studies. If no agreement can be achieved, a third researcher was added.

### Inclusion criteria

We chose to evaluate the literature of PS reports published after 1980, considering the possible availability of computed tomography (CT) examinations.

### Exclusion criteria

We excluded articles that did not report anagraphic data for each patient, the diagnostic method, the treatment modality, and the outcome for each case. The following data was collected from the included studies: correspondent author specialization, publication year, age, sex, duration of symptoms, pretreatment diagnosis modalities, treatment modalities, outcome (healed-improved/nonhealed) follow-up, intraoperative findings.

The final update of the literature search was conducted in December 2024.

### Statistical analysis

All data were initially entered into an Excel database (Microsoft, Redmond, Washington, United States) and the analysis was performed using the Statistical Package for the Social Sciences for Windows, version 21.0 (SPSS IBM Corp., Armonk, NY). Descriptive statistics consisted of the mean ± standard deviation [mean ± sd] for parameters with Gaussian distributions (after confirmation with histograms and the Kolmogorov‒Smirnov test) and the median and range of the 1 st and 3rd interquartiles [median (1°int.q; 3°int.q)] for nonGaussian parameters.

Unidirectional ANOVA was used for comparisons between normal data and the chi-square test ((2) or Fisher’s exact test (if the number of cells was < 5) for frequency data and the median test for non-normal parameters. A value of *p* < 0.05 was considered statistically significant.

## Results

**Fig. 1:**
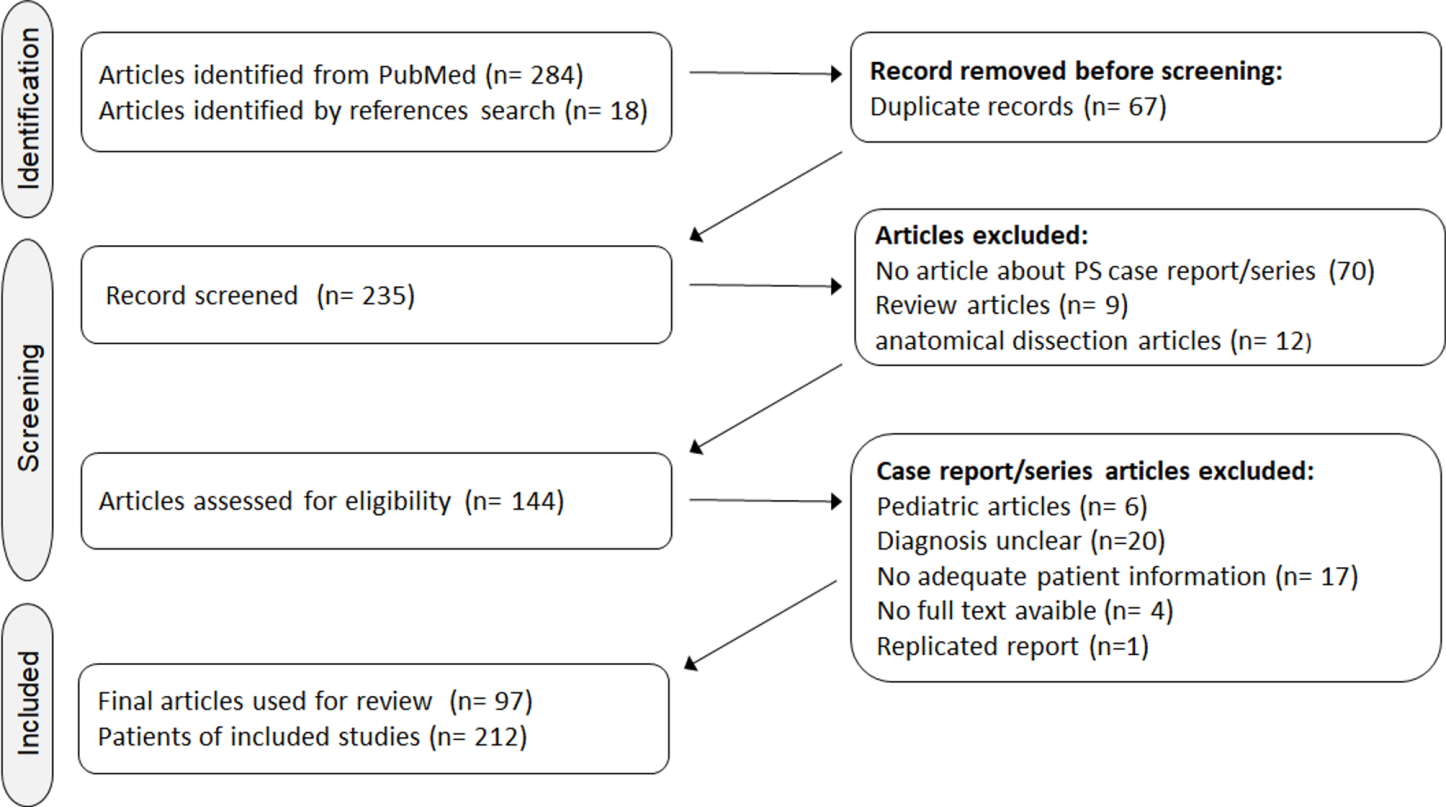
The preferred reporting items of systematic reviews guidelines flow diagram

A total of 302 articles were identified using PubMed (284) and manual reference search (18) (Fig. 1). After excluding 67 duplicate articles, 235 were reviewed by title and abstract. 70 articles were excluded because they do not focus on PS/PMs case report/series, 9 were review articles, and 12 were articles on anatomical dissection. 144 articles were considered for eligibility. From those we excluded 6 pediatric articles (under 15 years of age), 20 articles with unclear diagnosis, 16 with no adequate patient information, 4 because there is no full text available, 1 replicated report.

Finally, we used for review 97 articles (Table 1) that reported data from 212 patients [117 females (42.2 ± 13.8 age; range (15;72) age) and 95 males (45.3 ± 15.8 age; range (15;74) age)] with a homogeneous average age (Anova; *p* = 0,128). The median duration of symptoms before treatment was 365 days (60;1095) and the median follow-up period was 540 days (180;1140).

**Table 1:** Grouped data on the diagnosis and treatment modalities reported in the included articles

Authors, year	(F; *N*;age/M; *N*;age)*	Diag.^	Srg.°	Authors, year	(F; *N*;age/M; *N*;age)*	Diag.^	Srg.°
Adams,1980 [10]	(F:1:44,0/M:3:46,7)	Clin.	Y	Domínguez-Páez et al.,2012 [59]	(F:1:29,0/M:0:0,0)	instr.	Y
Solheim et al.,1981 [11]	(F:2:32,0/M:0:0,0)	Clin.	Y	Giebaly et al.,2012 [60]	(F:0:0,0/M:1:18,0)	Clin.	N
Augustin et al.,1984 [12]	(F:0:0,0/M:1:69,0)	instr.	Y	Kitagawa et al.,2012 [61]	(F:0:0,0/M:1:62,0)	instr.	Y
Karl et al.,1985 [13]	(F:0:0,0/M:1:41,0)	instr.	N	Wong et al.,2012 [62]	(F:1:31,0/M:0:0,0)	instr.	N
Papadopoulos,1990 [14]	(F:1:40,0/M:0:0,0)	instr.	Y	de la Peña Parra et al.,2013 [63]	(F:0:0,0/M:1:34,0)	Clin.	N
Barton,1991 [15]	(F:2:35,0/M:2:36,0)	3Clin./1instr.	N	Hamdi et al.,2013 [64]	(F:1:60,0/M:0:0,0)	instr.	N
Durrani et al.,1991 [16]	(F:0:0,0/M:1:41,0)	Clin.	N	Koda et al.,2013 [65]	(F:1:42,0/M:0:0,0)	instr.	Y
Jankiewicz et al.,1991 [17]	(F:1:27,0/M:0:0,0)	instr.	N	Polesello et al.,2013[66]	(F:1:42,0/M:0:0,0)	instr.	Y
Park et al.,1991 [18]	(F0:0,0/M:1:62,0)	instr.	Y	Sivrioglu et al.,2013 [67]	(F:1:27,0/M:0:0,0)	instr.	N
Vandertop,1991 [19]	(F:0:0,0/M:1:51,0)	Clin.	Y	Arooj et al.,2014 [68]	(F:1:35,0/M:0:0,0)	instr.	Y
Chen,1992 [20]	(F:0:0,0/M:1:42,0)	instr.	Y	Menu et al.,2014 [69]	(F:0:0,0/M:2:25,0)	Clin.	N
Hughes et al.,1992 [21]	(F:3:42,0/M:2:39,3)	instr.	Y	Ortiz Sánchez et al.,2014 [70]	(F:1:42,0/M:0:0,0)	Clin.	Y
Lam et al.,1993 [22]	(F:0:0,0/M:1:51,0)	instr.	Y	Ozisik et al.,2014 [71]	(F:8:51,1/M:2:64,5)	Clin.	N
Picco,1993 [23]	(F:1:29,0/M:0:0,0)	instr.	Y	Parlak et al.,2014 [72]	(F:0:0,0/M:1:41,0)	instr.	N
Sayson et al.,1994 [24]	(F:1:38,0/M:0:0,0)	Clin.	Y	Drampalos et al.,2015[73]	(F:1:48,0/M:0:0,0)	instr.	Y
Wun-Schen,1994 [25]	(F:1:28,0/M:0:0,0)	instr.	Y	Haghnegahdar,2015 [74]	(F:1:28,0/M:0:0,0)	instr.	Y
Kinahan et al.,1995 [26]	(F:1:22,0/M:0:0,0)	instr.	N	Kulkarni et al.,2015 [7]	(F:0:0,0/M:1:60,0)	instr.	N
Kouvalchouk et al.,1996 [27]	(F:0:0,0/M:3:37,0)	instr.	Y	Moon et al.,2015 [75]	(F:1:32,0/M:0:0,0)	instr.	Y
Beauchesne et al.,1997 [28]	(F:0:0,0/M:1:32,0)	instr.	Y	Santamato et al.,2015 [76]	(F:0:0,0/M:1:55,0)	Clin.	N
Merlo et al.,1997 [29]	(F:0:0,0/M:1:33,0)	Clin.	Y	Villano et al.,2015 [8]	(F:1:70,0/M:0:0,0)	Clin.	N
Chusid et al.,1998 [30]	(F:0:0,0/M:1:17,0)	instr.	N	Yang et al.,2015 [77]	(F:0:0,0/M:1:64,0)	instr.	N
Hanania et al.,1998 [31]	(F:2:64,5/M:4:56,3)	Clin.	N	Yıldırım et al.,2015 [78]	(F:1:51,0/M:0:0,0)	instr.	N
Benson et al.,1999 [32]	(F:9:36,2/M:5:42,0)	Clin.	Y	Zeren et al.,2015 [79]	(F:0:0,0/M:2:29,0)	instr.	Y
Ozaki et al.,1999 [33]	(F:1:22,0/M:0:0,0)	instr.	Y	Kraus et al.,2016 [80]	(F:0:0,0/M:1:68,0)	instr.	Y
Rossi et al.,2001 [34]	(F:1:30,0/M:0:0,0)	instr.	N	Vas et al.,2016 [81]	(F:0:0,0/M:1:72,0)	Clin.	N
Spinner et al.,2001 [35]	(F:1:44,0/M:0:0,0)	Clin.	Y	Han et al.,2017 [82]	(F:8:60,5/M:4:62,0)	7Clin./5instr.	Y
Foster,2002 [36]	(F:5:44,6/M:2:57,0)	instr.	Y	Phadke,2017 [83]	(F:0:0,0/M:1:21,0)	instr.	Y
Indrekvam et al.,2002 [37]	(F:15:41,9/M:4:47,0)	Clin.	Y	Wada et al.,2017 [84]	(F:0:0,0/M:1:53,0)	instr.	Y
Burkhart et al.,2003 [38]	(F:0:0,0/M:1:69,0)	instr.	N	Fusco et al.,2018 [85]	(F:2:55,0/M:1:55,0)	2Clin./1instr.	N
Jroundi et al.,2003 [39]	(F:1:30,0/M:0:0,0)	instr.	N	Ripellino et al.,2019 [86]	(F:0:0,0/M:1:35,0)	instr.	N
Nakamura et al.,2003 [40]	(F:1:40,0/M:1:23,0)	instr.	Y	Aquino-Jose et al.,2020 [87]	(F:1:36,0/M:1:29,0)	Clin.	N
Chong et al.,2004 [41]	(F:1:30,0/M:0:0,0)	instr.	N	Fahmi et al.,2020 [88]	(F:0:0,0/M:1:72,0)	Clin.	Y
Guyomarc’h et al.,2004 [42]	(F:1:38,0/M:2:26,0)	2Clin./1instr.	N	Hogan et al.,2020 [89]	(F:3:53,3/M:0:0,0)	1Clin./2instr.	Y
Lee et al.,2004 [43]	(F:0:0,0/M:1:40,0)	instr.	Y	Koh et al.,2020 [90]	(F:0:0,0/M:1:18,0)	instr.	N
Vallejo et al.,2004 [44]	(F:1:29,0/M:0:0,0)	instr.	N	Leong et al.,2020 [91]	(F:0:0,0/M:1:24,0)	instr.	N
Hettler et al.,2006 [45]	(F:1:44,0/M:0:0,0)	instr.	Y	Ou Yang et al.,2020 [92]	(F:1:16,0/M:0:0,0)	instr.	Y
Kosukegawa et al.,2006 [46]	(F:0:0,0(M:1:57,0)	instr.	Y	Akbas et al.,2021 [93]	(F:1:18,0/M:0:0,0)	instr.	Y
Turtas et al.,2006 [47]	(F:0:0,0/M:1:60,0)	instr.	Y	Kale et al.,2021 [94]	(F:1:42,0/M:0:0,0)	instr.	Y
Colmegna et al.,2007 [48]	(F:1:18,0/M:0:0,0)	instr.	N	Lodin et al.,2021 [95]	(F:0:0,0/M:1:55,0)	instr.	Y
Kabataş et al.,2008 [49]	(F:1:36,0/M:0:0,0)	Clin.	N	Salehi et al.,2021[96]	(F:0:0,0/M:1:43,0)	instr.	N
Kobbe et al.,2008 [50]	(F:0:0,0/M:2:45,0)	instr.	Y	Shanmuga Jayanthan et al.,2021 [97]	(F:0:0,0/M:1:41,0)	instr.	Y
Wong et al.,2008 [51]	(F:3:58,0/M:0:0,0)	instr.	N	Chua et al.,2022 [98]	(F:1:63,0/M:0:0,0)	instr.	N
Dere et al.,2009 [52]	(F:2:27,5/M:0:0,0)	instr.	N	Güleç et al.,2022 [99]	(F:1:30,0/M:3:30,6)	instr.	N
Niu et al. 2009 [53]	F:6:43,0/M:6:46,6)	Clin.	N	Kaga et al.,2022 [100]	(F:0:0,0/M:1:71,0)	Clin.	N
Yoshimoto et al.,2009 [54]	(F:2:55,0/M:1:59,0)	2Clin./1instr.	2Y/1 N	Kwon et al.,2022 [101]	(F:0:0,0/M:1:40,0)	instr.	N
Hwang et al.,2010 [55]	(F:1:42,0/M:0:0,0)	instr.	Y	Ward et al.,2022 [102]	(F:0:0,0/M:1:14,0)	instr.	Y
Jawish et al.,2010 [56]	(F:4:28,5/M:5:41,8)	1Clin./8instr.	Y	Gebregiorigis et al.,2024 [103]	(F:0:0,0/M:1:14,0)	instr.	Y
Jeon et al.,2010 [57]	(F:1:50,0/M:0:0,0)	instr.	N	Qiu et al.,2024 [104]	(F:0:0,0/M:1:14,0)	instr.	Y
Ye et al.,2010 [58]	(F.0:0,0/M:1:74,0)	instr.	N				

(*) F;N; age/M;N; age= Females; Number; age/Males; Number; age

(^) *Diag.* Diagnosis, *Clin. *Clinical Diagnosis, *instr. *Diagnosis corroborated by instrumental examination or PM injection test

(°) Srg.= Surgery; Yes= Y; No= *N*

### Medical history

15.1% (32/212) of the patients practised sports involving the lower limbs; 7 cycling, 7 runners, 7 soccer, 4 gymnastics/fitness, 2 tennis, 2 swimming, 2 australian football/rugby, 1 basketball.

A total of 38.2% (81/212) of the patients in this study had a history of blunt trauma (e.g. falling to the bottom) or indirect pelvic trauma (e.g. hip sprain), PM stress due to vigorous physical activity (e.g. snow shoveling) or sport practice. A total of 4.7% (10/212) of the patients reported factors associated with pyomyositis/hematoma: 4 patients with gynecological surgical treatment/pathology, such as partum epidural analgesia and abort, 2 patients with anticoagulant treatment, 2 patients with postinjection gluteal abscess, 1 patient with recent lung infection, and 1 patient with hydronephrosis.

In 57.1% (121/212) of the patients, the authors did not report any factors associated with PS.

### Diagnosis

**Fig. 2:**
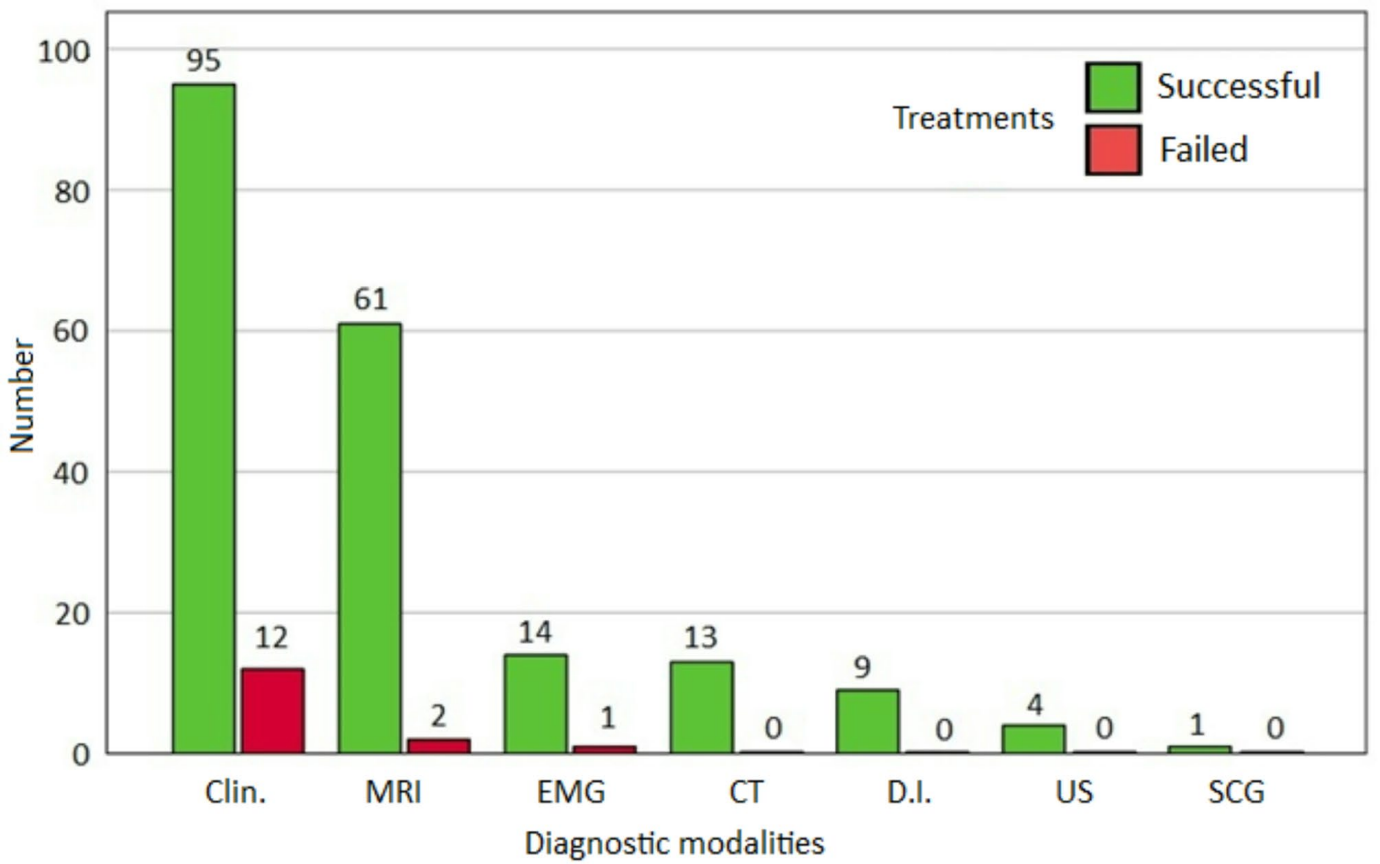
Number of patients who had successful/failed treatments in relation to the diagnostic modalities

Before treatment, our review revealed that 49.5% (105/212) of the patients had PS/PMs diagnosis corroborated by an instrumental exam or PM diagnostic injections (D.I.). The diagnosis of PS/PMs was corroborated in detail as follows: 29.7% (63/212) by intrapelvic MRI or magnetic resonance neurography, 2 of whom did not heal; 7.1% (15/212) only by electromyography (EMG), 6 of them had some improvement after PM injection before surgery, 1 did not heal. 6.1% (13/212) by CT, 4.2% (9/212) only by PM diagnostic injections (D.I.), 1.9% (4/212) by diagnostic ultrasound (US), and 0.5% (1/212) by bone scintigraphy (SCG), all patients healed. A total of 50.5% (107/212) of the patients were diagnosed with PS by the symptoms, medical history, and P.E. (clinical diagnosis), 12 of whom did not heal. The graph in Fig. 2 shows the number of patients who had successful/failed treatment compared to the diagnostic modalities.

After treatment, according to the author’s conclusions, by instrumental examination or intraoperative finding, the etiological factors of PS/PMs were as follows (Table 2): 12.7% (27/212) PM or SN anatomical variant, 9.4% (20/212) PM pyomyositis, 7.5% (16/212) PM hypertrophy, 5.7% (12/212) fibrous adherent scar tissue around the SN, 3.8% (8/212) vascular anomalies (e.g. plexus of veins surrounding the SN, loop in a collateral of the inferior gluteal artery compressing the SN, pseudoaneurysm, aneurysm), 1,9% (4/212) PM hematoma, 0.9% (2/212) lipoma 0.9% (2/212) endometriosis, 0.5% (1/212) PM atrophy, PM calcification, 0.5% (1/212) cyst impinging SN, 0.5% (1/212) PM lymphoma 0.5% (1/212), PM myositis ossificans 0.5% (1/212). Furthermore, in other patients, the authors reported 3.8% (8/212) PM/SN MRI hyperintensity, 1.4% (3/212) EMG findings of SN H reflex delay/absence, 0.9% (2/212) PM/SN US hyperechoic signal, 0.5% (1/212) SCG findings of abnormal PM uptake. 47.6% (101/212) of the patients had no instrumental or intraoperative findings.

### Treatment modalities

40.1% (85/212) of the patients received nonsurgical treatment: 25.0% (53/212) received local injection with anesthetic and/or corticosteroids, 5.7% (12/212) received antibiotic therapy, 4.7% (10/212) received specific exercise programs and/or physiotherapy, 1.9% (4/212) received botulinum injection, 1.4% (3/212) received dry needling, 0.5% (1/212) received chemotherapy, 0.5% (1/212) received shockwave therapy, 0.5% (1/212) received drug suspension (atorvastatin).

Most of the patients (59.9% (127/212)) had surgical treatment. The surgical procedures used were as follows: 49.1% (104/212) release of the piriformis tendon (in the presence/absence of adhesions, vascular anomalies, PM pyomyositis, anatomical variant, hematoma, lipoma), 3.8% (8/212) neurolysis of the SN, 3.3% (7/212) surgical drainage of abscesses, 2.4% (5/212) masses resection (hematoma, lipoma, cyst, endometriosis), 0.9% (2/212) aneurysm resection and 0.5% (1/212) pseudoaneurysm decompression (Table 2).

**Table 2:** Treatment modalities and definitive instrumental and intraoperative findings

Treatment modalities	*n*	%
Piriformis tendon release	104	49.1
SN neurolysis	8	3.8
Abscess drainage	7	3.3
Masses resection	5	2.4
Aneurysm resection	2	0.9
Pseudoaneurysm decompression	1	0.5
Anesthetic/corticosteroids injection	53	25.0
Antibiotic therapy	12	5.7
Exercise programs/physiotherapy	10	4.7
Botulinum injection	4	1.9
Dry nilling	3	1.4
Chemotherapy	1	0.5
Shockwave therapy	1	0.5
Suspension of drug	1	0.5
Total	212	100.0

Definitive instrumental/intraoperative findings	*n*	%
PM/SN anatomical variant	27	12.7
PM pyomyositis	20	9.4
PM hypertrophy	16	7.5
Scar tissue	12	5.7
Vascular anomalies	8	3.8
PM hematoma	4	1.9
PM lipoma	2	0.9
PM endometriosis	2	0.9
PM atrophy	1	0.5
PM calcification	1	0.5
Cyst	1	0.5
PM lymphoma	1	0.5
PM myositis ossificans	1	0.5
PM/SN hyperintensity (MRI)	8	3.8
SN H reflex delay/absence (EMG)	3	1.4
PM/SN hyperechoic signal (US)	2	0.9
PM abnormal uptake (SCG)	1	0.5
Diffusion MRI tractography SN/PM findings	1	0.5
No instrumental/intraoperative findings	101	47.6
Total	212	100.0

### Outcome

In total, 197/212 (92.9%) patients healed/improved after therapy. In detail: 116/197 (58.9%) patients healed after surgery while 81/197 (41.1%) patients healed after nonsurgical therapy; oppositely, 15/212 (7.1%) patients had no pain relief (11/15 after surgery and 4/15 after nonsurgical treatment).

In 127 of 212 (59.9%) surgically treated patients, 69 of 127 (54.3%) had a diagnosis of PS corroborated by an instrumental exam/D.I., while 58 of 127 (45.7%) patients had a clinical diagnosis.

In the 69/127 group, only 2 (2.9%) patients did not heal after surgery, while in the 58/127 group, 9 (15.5%) patients did not heal: In the last group the OR of surgical treatment failure occurrence was (15.5%/2.9%) = 5.3; 95% Confidence Interval (CI) (5,0;5,8).

In 85 of 212 (40.1%) nonsurgically treated patients, 36 of 85 (42.4%) had a diagnosis of PS corroborated by instrumental examination, while 49 of 85 (57.6%) patients had a clinical diagnosis.

In the 36/85 group only 1 patient (2.8%) did not heal after conservative treatment, while in the 49/85 group 3 (6.1%) patients did not heal after treatment: the odds ratio (OR) was (6.1%/2.8%) = 2.2; 95% CI (2,1;2,4).

We also found that 9.0% (19/212) of the patients had previously failed lumbar spine surgery (14 females; 5 males): 17 of them had pain relief after PS therapy.

## Discussion

Our review specifically addressed the presumed or confirmed cases of SN compression involving the PM: We excluded other intrapelvic impingements of the SN [105, 106], which deserve a differential diagnosis.

Consistent with other studies [107–109] the patients included in this review were mainly women (female to male ratio of 1.2:1), with an average age of 43.6 years, in the age range of the highest risk of PS (30–50 years).

The medical history of PS patients often shows that an event is correlated with muscular damage. Direct or indirect pelvic trauma or strenuous physical exercise in PM can be associated with muscular damage. The resulting soft tissue inflammatory changes and edema can predispose individuals to SN impingement or scarring surrounding the SN, thus causing SN entrapment [110]. In our review, 38.2% of patients reported direct or indirect pelvic trauma or PM stress due to vigorous physical activity/sport practice.

In general, the authors considered the positivity of the following tests and signs a P.E. suggestive of PS [6, 36, 64]: the piriformis sign (a persistent external rotation of the extended limb in the supine patient); the Freiberg test (sciatica appears with forceful internal rotation of the hip with the patient supine); the Beatty test (sciatica appears when the patient maintains the hip flexed in abduction against gravity while lying on the non -affected side); the Pace test (buttock pain increases by applying resistance to the abduction of the hip holding the patient’s knee in the sitting position); the FAIR position (pain is exacerbated with the hip flexed, adducted, and internally rotated).

PM pyomyositis in its early appearance causes sciatica and represents a fearful cause of PS because delay in diagnosis can be life-threatening [50]. The clinical signs of PM pyomyositis are typically mild buttock pain and sciatica for a few days, followed by an increase in body temperature [89]. Its pathogenesis may be related to muscle damage with concomitant asymptomatic bacteremia [38, 91]. Specifically, Staphylococcus is the microorganism most often isolated from patients. In our review, blood cultures also yielded P. mirabilis Group B Streptococcus and Salmonella typhi. In three cases, the infectious agent has not been identified [26, 40, 64].

For all cases of PM pyomyositis reported here (20 cases), MRI or CT scans allowed rapid diagnosis and therapy. In 4 cases, pyomyositis occurred in patients who practice sports [30, 38, 59, 91].

MRI scan of the pelvis is the most commonly adopted test to corroborate the diagnosis of PS/PMs (29.7%).

A pelvic MRI neurogram with complex oblique planes may reveal anatomical variants of SN [79].

The diffusion MR tractography technique allows the orientation of the nerve fibers to be followed for tracing specific neural pathways and visualizing the condition of the nerve fibers [84].

A standard MRI scan can detect localized or diffuse changes in the PM, a mass impinging the SN, but sometimes it can identify only an increase in PM volume [17].

In contrast to the theory of an association between PM hypertrophy and PS, some authors believe that PM asymmetry is common in asymptomatic subjects [32].

In this review, PS associated with PM hypertrophy was treated with good results in 11 out of 13 patients.

In some patients, diagnostic imaging is unblemished, but EMG can reveal findings suggesting sciatic nerve entrapment in PM [21].

Ozisik et al. [70] reported that intrapelvic sciatic nerve impingement should delay the H reflex. In PS patients, EMG under standard conditions may not reveal pathological findings. PM stimulation may induce changes in EMG: Zeren et al. [78], in two male soccer players, reported pathological changes in a previously normal EMG after a short run or by placing the affected lower limb in internal rotation and adduction (pain position).

Nakamura et al. [40] detect the potential of the cauda equina using an epidural electrode. The potentials were recorded first with the patient’s leg extended and then with the patient’s hip flexed and rotated internally: In this posture, on the affected side, the recorded potentials exhibited polyphasic deformity.

After a negative or nonspecific instrumental diagnostic assessment, some authors perform a D.I. and make a diagnosis of PS based on the temporary disappearance of symptoms [36, 82].

Local injection of drugs to treat PS could have adverse effects, such as anesthetic intoxication, infection, and muscle atrophy after steroid injection, and weakness and pain after botulinum toxin injection [100]. In this review, no adverse effects were reported.

The most frequent diagnosis of PS/PMS has been identified in a PM or SN anatomical variant (12.3%) followed by PM pyomyositis (6.1%), PM hypertrophy (5.7%). In some cases, even intraoperative inspection could not identify any cause of SN impingement [56].

In one patient, an MRI scan revealed PS caused by statin-induced piriformis myopathy: patient recovery occurred after therapy suspension [64]. He previously received an ineffective local corticosteroids injection.

59.9% of patients underwent surgery; complications of SN entrapment surgery include hematoma formation, infections, permanent paresthesia, hyperesthesia, superficial surgical site infection, deep wound infection, failure to resolve pain or worsened pain [111–113]. Justice et al. [111] reported two cases of permanent impairment of muscle function below the knee after PM release surgery (paper not included in our database). The injury mechanism hypothesized for this serious complication was blade retraction, as occurs in prosthetic hip surgery. In our review, in three patients, recurrence occurred a few months after PM release and these patients underwent revision surgery [50, 74].

Surgery did not relieve pain in 11 patients [35, 37, 56, 74, 82]. Nine of them had no instrumental diagnosis nor a positive D.I. was reported: in the group of patients with PS clinical diagnosis, the OR of surgical failure occurrence was 5.3.

To avoid unnecessary spine surgery, Niu et al. [53] suggest excluding PS before diagnosing lumbar radiculopathy: In this review, 9% (19/212) of the patients had failed spine surgery and 89.5% of them (17) had pain relief after PS treatment.

### Study limitations

Each physician or surgeon adopted their own diagnostic protocol. In cases of suspected PS, priority was given to different diagnostic modalities — for instance, DI was sometimes performed before EMG or MRI, and vice versa. This variability made it difficult to compare the effectiveness of individual diagnostic tools across similar cases.

The limitations of this review also include case report admission and selection bias: case reports are usually selected for publication because outliers are unique. Furthermore, the natural tendency of authors and journals to propose and publish positive cases could influence the overall outcome, increasing the number of cases with good results [114, 115].

Unfortunately, it was not possible to describe the anthropometric data (weight, height) of the patients due to the lack of such information in the articles.

## Conclusions

Intrapelvic MRI was the instrumental examination most frequently used to confirm the diagnosis of PS/PMs prior to treatment. After treatment, the causes most frequently identified by instrumental exam or intraoperative finding were the anatomical variant of PM or SN, PM pyomyositis, and PM hypertrophy.

In the group of patients with PS clinical diagnosis the OR of surgical treatment failure occurrence was 5.3. Given the risks that sciatic nerve surgery in this region may entail, underestimated by this review, in suspected PS, it is needed to carry out all available instrumental diagnostic procedures to reduce surgical failures and complications.

## Supplementary Information

Supplementary Material 1.

## Data Availability

Data are available from the corresponding author on reasonable request.

## Abbreviations

SCG: Bone scintigraphy
CT: Computed tomography
CI: Confidence interval
Clin.: Clinical diagnosis
D.I.: Diagnostic injections
Diag.: Diagnosis
EMG: Electromyography
instr.: Diagnosis corroborated by instrumental examination or PM injection test
MRI: Magnetic resonance imaging
OR: Odds ratio
PM: Piriformis muscle
PMs: Piriformis muscle sciatica
PS: Piriformis syndrome
P.E.: Physical examination
SN: Sciatic nerve
Srg.: Surgery
US: Ultrasound

## References

[1] Stafford MA, Peng P, Hill DA. Sciatica: a review of history, epidemiology, pathogenesis, and the role of epidural steroid injection in management. Br J Anaesth. 2007;99(4):461–73. 17704089 10.1093/bja/aem238

[2] Ropper AH, Zafonte RD, Sciatica. Longo DL, editors. N Engl J Med. 2015;372(13):1240–8. 10.1056/NEJMra1410151 25806916

[3] Robinson DR. Pyriformis syndrome in relation to sciatic pain. Am J Surg. 1947;73(3):355–8. 20289074 10.1016/0002-9610(47)90345-0

[4] International Statistical Classification of Diseases and Related Health (ICD-11.). https://icd.who.int/ct/icd11_mms/en/release. Accessed 13 Sep 2024.

[5] Hicks BL, Lam JC, Varacallo M, Piriformis S. In: StatPearls. Treasure Island (FL): StatPearls Publishing; 2024 Jan–. 4 Aug 2023. PMID: 28846222.

[6] Hopayian K, Danielyan A. Four symptoms define the piriformis syndrome: an updated systematic review of its clinical features. Eur J Orthop Surg Traumatol. 2018;28(2):155–64. 28836092 10.1007/s00590-017-2031-8

[7] Kulkarni R, Borole B, Chaudhary J, Dev S. A case of piriformis syndrome presenting as radiculopathy. Indian J Pain. 2015;29(2):115.

[8] Villano EQ, Rijhwani K. A case of piriformis syndrome mimicking radiculopathy. Journal on Recent Advances in Pain. 2015;1(1):24–5.

[9] Jackson D, Daly J, Saltman DC. Aggregating case reports: a way for the future of evidence-based health care? Clin Case Rep. 2014;2(2):23–4. 10.1002/ccr3.58. PMID: 25356237; PMCID: PMC4184623. 25356237 10.1002/ccr3.58 PMC4184623

[10] Adams JA. The pyriformis syndrome -- report of four cases and review of the literature. S Afr J Surg. 1980;18(1):13–8. PMID: 7384951. 7384951

[11] Solheim LF, Siewers P, Paus B. The piriformis muscle syndrome. Sciatic nerve entrapment treated with section of the piriformis muscle. Acta Orthop Scand. 1981;52(1):73–5. 10.3109/17453678108991762. PMID: 6452020.12.Augustin et al 1984.pdf. 6452020 10.3109/17453678108991762

[12] Augustin P, Daluzeau N, Dujardin M, Clement O, Denis P. Hématome du muscle pyramidal. Complication d’un traitement anti-coagulant [Hematoma of the pyramidal muscle. A complication of anticoagulant treatment]. Rev Neurol (Paris). 1984;140(6–7):443–5. French. PMID: 6235567. 6235567

[13] Karl RD Jr, Yedinak MA, Hartshorne MF, Cawthon MA, Bauman JM, Howard WH, Bunker SR. Scintigraphic appearance of the piriformis muscle syndrome. Clin Nucl Med. 1985;10(5):361–3. 10.1097/00003072-198505000-00011. PMID: 3160520. 3160520 10.1097/00003072-198505000-00011

[14] Papadopoulos SM, McGillicuddy JE, Albers JW. Unusual cause of ‘piriformis muscle syndrome.’ Arch Neurol. 1990;47(10):1144–6. 10.1001/archneur.1990.00530100114027. PMID: 2222250. 2222250 10.1001/archneur.1990.00530100114027

[15] Barton PM. Piriformis syndrome: a rational approach to management. Pain. 1991;47(3):345–52. 10.1016/0304-3959(91)90227-O. PMID: 1784505kou. 1784505 10.1016/0304-3959(91)90227-O

[16] Durrani Z, Winnie AP. Piriformis muscle syndrome: an underdiagnosed cause of sciatica. J Pain Symptom Manage. 1991;6(6):374–9. 10.1016/0885-3924(91)90029-4. PMID: 1880438. 1880438 10.1016/0885-3924(91)90029-4

[17] Jankiewicz JJ, Hennrikus WL, Houkom JA. The appearance of the piriformis muscle syndrome in computed tomography and magnetic resonance imaging. A case report and review of the literature. Clin Orthop Relat Res. 1991;(262):205–9. PMID: 1984918. 1984918

[18] Park HW, Jahng JS, Lee WH. Piriformis syndrome–a case report. Yonsei Med J. 1991;32(1):64–8. 10.3349/ymj.1991.32.1.64. PMID: 1877257. 1877257 10.3349/ymj.1991.32.1.64

[19] Vandertop WP, Bosma NJ. The piriformis syndrome. A case report. J Bone Joint Surg Am. 1991;73(7):1095–7. PMID: 1874775. 1874775

[20] Chen WS. Sciatica due to piriformis pyomyositis. Report of a case. J Bone Joint Surg Am. 1992;74(10):1546–8. PMID: 1469016. 1469016

[21] Hughes SS, Goldstein MN, Hicks DG, Pellegrini VD Jr. Extra pelvic compression of the sciatic nerve. An unusual cause of pain about the hip: report of five cases. J Bone Joint Surg Am. 1992;74(10):1553–9. PMID: 1469018. 1469018

[22] Lam AW, Thompson JF, McCarthy WH. Unilateral piriformis syndrome in a patient with previous melanoma. Aust N Z J Surg. 1993;63(2):152–3. 10.1111/j.1445-2197.1993.tb00067.x. PMID: 8297307. 8297307 10.1111/j.1445-2197.1993.tb00067.x

[23] Picco AG, Parajua Pozo JL. Síndrome Del músculo piriforme Por piomiositis [The piriformis muscle syndrome due to pyomyositis]. Med Clin (Barc). 1993;100(11):436–7. Spanish. PMID: 7639820. 7639820

[24] Sayson SC, Ducey JP, Maybrey JB, Wesley RL, Vermilion D. Sciatic entrapment neuropathy associated with an anomalous piriformis muscle. Pain. 1994;59(1):149–52. 10.1016/0304-3959(94)90060-4. PMID: 7854796. 7854796 10.1016/0304-3959(94)90060-4

[25] Wun-Schen C. Bipartite piriformis muscle: an unusual cause of sciatic nerve entrapment. Pain. 1994;58(2):269–72. 10.1016/0304-3959(94)90208-9. PMID: 7816495. 7816495 10.1016/0304-3959(94)90208-9

[26] Kinahan AM, Douglas MJ. Piriformis pyomyositis mimicking epidural abscess in a parturient. Can J Anaesth. 1995;42(3):240–5. 10.1007/BF03010686. PMID: 7743579. 7743579 10.1007/BF03010686

[27] Kouvalchouk JF, Bonnet JM, de Mondenard JP. Le syndrome du pyramidal. A Propos de 4 Cas traités chirurgicalement et revue de La littérature [Pyramidal syndrome. Apropos of 4 Cases treated by surgery and review of the literature]. Rev Chir Orthop Reparatrice Appar Mot. 1996;82(7):647–57. French. PMID: 9091984. 9091984

[28] Beauchesne RP, Schutzer SF. Myositis ossificans of the piriformis muscle: an unusual cause of piriformis syndrome. A case report. J Bone Joint Surg Am. 1997;79(6):906–10. 10.2106/00004623-199706000-00016. PMID: 9199390. 9199390 10.2106/00004623-199706000-00016

[29] Merlo LM, Poloni TE, Alfonsi E, Messina AL, Ceroni M. Sciatic pain in a young sportsman. Lancet. 1997;349(9055):846. 9121261 10.1016/s0140-6736(97)01120-3

[30] Chusid MJ, Hill WC, Bevan JA, Sty JR. Proteus pyomyositis of the piriformis muscle in a swimmer. Clin Infect Dis. 1998;26(1):194–5. 10.1086/517062. PMID: 9455539. 9455539 10.1086/517062

[31] Hanania M, Kitain E. Perisciatic injection of steroid for the treatment of sciatica due to piriformis syndrome. Reg Anesth Pain Med. 1998;23(2):223–8. 10.1097/00115550-199823020-00020. PMID: 9570616. 9570616 10.1097/00115550-199823020-00020

[32] Benson ER, Schutzer SF. Posttraumatic piriformis syndrome: diagnosis and results of operative treatment. J Bone Joint Surg Am. 1999;81(7):941–9. PMID: 10428125. 10428125

[33] Ozaki S, Hamabe T, Muro T. Piriformis syndrome resulting from an anomalous relationship between the sciatic nerve and piriformis muscle. Orthopedics. 1999;22(8):771-2. 10.3928/0147-7447-19990801-09. PMID: 10465490. 10465490 10.3928/0147-7447-19990801-09

[34] Rossi P, Cardinali P, Serrao M, Parisi L, Bianco F, De Bac S. Magnetic resonance imaging findings in piriformis syndrome: a case report. Arch Phys Med Rehabil. 2001;82(4):519–21. 10.1053/apmr.2001.21971. PMID: 11295014. 11295014 10.1053/apmr.2001.21971

[35] Spinner RJ, Thomas NM, Kline DG. Failure of surgical decompression for a presumed case of piriformis syndrome. J Neurosurg. 2001;94(4):652–4. 10.3171/jns.2001.94.4.0652. PMID: 11302670. 11302670 10.3171/jns.2001.94.4.0652

[36] Foster MR. Piriformis syndrome. Orthopedics. 2002;25(8):821–5. 10.3928/0147-7447-20020801-12. PMID: 12195908. 12195908 10.3928/0147-7447-20020801-12

[37] Indrekvam K, Sudmann E. Piriformis muscle syndrome in 19 patients treated by tenotomy–a 1- to 16-year follow-up study. Int Orthop. 2002;26(2):101–3. 10.1007/s00264-001-0319-z. PMID: 12078870; PMCID: PMC3620867. 12078870 10.1007/s00264-001-0319-z PMC3620867

[38] Burkhart BG, Hamson KR. Pyomyositis in a 69-year-old tennis player. Am J Orthop (Belle Mead NJ). 2003;32(11):562-3. PMID: 14653488. 14653488

[39] Jroundi L, El Quessar A, Chakir N, El Hassani MR, Jiddane M. Le syndrome du muscle pyramidal: Une cause rare de sciatique Non discale [The piriformis syndrome: a rare cause of Non-discogenic sciatica. A case report]. J Radiol. 2003;84(6):715–7. French. PMID: 12910180. 12910180

[40] Nakamura H, Seki M, Konishi S, Yamano Y, Takaoka K. Piriformis syndrome diagnosed by cauda equina action potentials: report of two cases. Spine (Phila Pa 1976). 2003;28(2):E37-40. 10.1097/00007632-200301150-00022. PMID: 12544943. 12544943 10.1097/00007632-200301150-00022

[41] Chong KW, Tay BK. Piriformis pyomyositis: a rare cause of sciatica. Singap Med J. 2004;45(5):229–31. PMID: 15143360. 15143360

[42] Guyomarc’h F, Labanere C. Syndrome du muscle piriformis: Un diagnostic différentiel de sciatalgie Chez Le sportif? À Propos de 3 Cas et revue de La littérature. J De Traumatologie Du Sport. 2004;21(3):133–45.

[43] Lee EY, Margherita AJ, Gierada DS, Narra VR. MRI of piriformis syndrome. AJR Am J Roentgenol. 2004;183(1):63–4. 10.2214/ajr.183.1.1830063. PMID: 15208111. 15208111 10.2214/ajr.183.1.1830063

[44] Vallejo MC, Mariano DJ, Kaul B, Sah N, Ramanathan S. Piriformis syndrome in a patient after cesarean section under spinal anesthesia. Reg Anesth Pain Med. 2004 Jul-Aug;29(4):364-7. 10.1016/j.rapm.2004.01.014. PMID: 15305258. 15305258 10.1016/j.rapm.2004.01.014

[45] Hettler A, Böhm J, Pretzsch M, von Salis-Soglio G. Piriformissyndrom infolge einer extragenitalen Endometriose [Extragenital endometriosis leading to piriformis syndrome]. Nervenarzt. 2006;77(4):474-7. German. 10.1007/s00115-005-2028-0. German. PMID: 16425055. 16425055 10.1007/s00115-005-2028-0

[46] Kosukegawa I, Yoshimoto M, Isogai S, Nonaka S, Yamashita T. Piriformis syndrome resulting from a rare anatomic variation. Spine (Phila Pa 1976). 2006;31(18):E664-6. 10.1097/01.brs.0000231877.34800.71. PMID: 16915082. 16915082 10.1097/01.brs.0000231877.34800.71

[47] Turtas S, Zirattu G. The piriformis syndrome: a case report of an unusual cause of sciatica. J Orthopaed Traumatol. 2006;7:97–9. 10.1007/s10195-006-0129-6.

[48] Colmegna I, Justiniano M, Espinoza LR, Gimenez CR. Piriformis pyomyositis with sciatica: an unrecognized complication of unsafe abortions. J Clin Rheumatol. 2007;13(2):87–8. 10.1097/01.rhu.0000260655.90449.7d. PMID: 17414537. 17414537 10.1097/01.rhu.0000260655.90449.7d

[49] Kabataş S, Gümüş B, Yilmaz C, Caner H. CT-guided corticosteroid injection as a therapeutic management for the pyriformis syndrome: case report. Turk Neurosurg. 2008;18(3):307–10. PMID: 18814124. 18814124

[50] Kobbe P, Zelle BA, Gruen GS. Case report: recurrent piriformis syndrome after surgical release. Clin Orthop Relat Res. 2008;466(7):1745–8. 10.1007/s11999-008-0151-5. Epub 2008 Feb 9. PMID: 18264837; PMCID: PMC2505267. 18264837 10.1007/s11999-008-0151-5 PMC2505267

[51] Wong CH, Choi SH, Wong KY. Piriformis pyomyositis: a report of three cases. J Orthop Surg (Hong Kong). 2008;16(3):389–91. 10.1177/230949900801600326. PMID: 19126914. 19126914 10.1177/230949900801600326

[52] Dere K, Akbas M, Luleci N. A rare cause of a piriformis syndrome. J Back Musculoskelet Rehabil. 2009;22(1):55–8. 10.3233/BMR-2009-0213. PMID: 20023365. 20023365 10.3233/BMR-2009-0213

[53] Niu CC, Lai PL, Fu TS, Chen LH, Chen WJ. Ruling out piriformis syndrome before diagnosing lumbar radiculopathy. Chang Gung Med J. 2009 Mar-Apr;32(2):182-7. PMID: 19403008. 19403008

[54] Yoshimoto M, Kawaguchi S, Takebayashi T, Isogai S, Kurata Y, Nonaka S, Oki G, Kosukegawa I, Yamashita T. Diagnostic features of sciatica without lumbar nerve root compression. J Spinal Disord Tech. 2009;22(5):328 – 33. 10.1097/BSD.0b013e31817dc46d. PMID: 19525787. 19525787 10.1097/BSD.0b013e31817dc46d

[55] Hwang DS, Kang C, Lee JB, Cha SM, Yeon KW. Arthroscopic treatment of piriformis syndrome by perineural cyst on the sciatic nerve: a case report. Knee Surg Sports Traumatol Arthrosc. 2010;18(5):681–4. 10.1007/s00167-009-1013-8. Epub 2010 Jan 9. PMID: 20062971. 20062971 10.1007/s00167-009-1013-8

[56] Jawish RM, Assoum HA, Khamis CF. Anatomical, clinical and electrical observations in piriformis syndrome. J Orthop Surg Res. 2010;5:3. 10.1186/1749-799X-5-3. PMID: 20180984; PMCID: PMC2828977. 20180984 10.1186/1749-799X-5-3 PMC2828977

[57] Jeon SY, Moon HS, Han YJ, Sung CH. Post-radiation piriformis syndrome in a cervical cancer patient -A case report-. Korean J Pain. 2010;23(1):88–91. 10.3344/kjp.2010.23.1.88. Epub 2010 Mar 10. PMID: 20552082; PMCID: PMC2884211. 20552082 10.3344/kjp.2010.23.1.88 PMC2884211

[58] Ye BS, Sunwoo IN, Suh BC, Park JP, Shim DS, Kim SM. Diffuse large B-cell lymphoma presenting as piriformis syndrome. Muscle Nerve. 2010;41(3):419–22. 10.1002/mus.21538. PMID: 19918770. 19918770 10.1002/mus.21538

[59] Domínguez-Páez M, de Miguel-Pueyo LS, Medina-Imbroda JM, González-García L, Moreno-Ramírez V, Martín-Gallego A, Socolovsky M, Arráez-Sánchez MÁ. Ciatalgia secundaria a endometriosis extrapélvica del músculo piriforme. A propósito de un caso [Sciatica secondary to extrapelvic endometriosis affecting the piriformis muscle. Case report]. Neurocirugia (Astur). 2012;23(4):170-4. Spanish. 10.1016/j.neucir.2012.04.009. Epub 2012 Jun 22. PMID: 22728121. 22728121 10.1016/j.neucir.2012.04.009

[60] Giebaly DE, Horriat S, Sinha A, Mangaleshkar S. Pyomyositis of the piriformis muscle presenting with sciatica in a teenage rugby player. BMJ Case Rep. 2012;2012: bcr1220115392. 10.1136/bcr.12.2011.5392. PMID: 22802567; PMCID: PMC3417030. 10.1136/bcr.12.2011.5392 PMC3417030 22802567

[61] Kitagawa Y, Yokoyama M, Tamai K, Takai S. Chronic expanding hematoma extending over multiple gluteal muscles associated with piriformis syndrome. J Nippon Med Sch. 2012;79(6):478–83. 10.1272/jnms.79.478. PMID: 23291848. 23291848 10.1272/jnms.79.478

[62] Wong LF, Mullers S, McGuinness E, Meaney J, O’Connell MP, Fitzpatrick C. Piriformis pyomyositis, an unusual presentation of leg pain post partum–case report and review of literature. J Matern Fetal Neonatal Med. 2012;25(8):1505–7. 10.3109/14767058.2011.636098. Epub 2011 Dec 6. PMID: 22082187. 22082187 10.3109/14767058.2011.636098

[63] de la Peña Parra E, Calle Romero Y, García Sánchez VC, Sanz Pozo B. Lumbalgia de evolución tórpida [Low back pain of unfavourable progression]. Semergen. 2013 Nov-Dec;39(8):453-5. Spanish. 10.1016/j.semerg.2012.07.005. Epub 2012 Sep 13. PMID: 24315078. 24315078 10.1016/j.semerg.2012.07.005

[64] Hamdi W, Ghannouchi MM, Kaffel D, Kchir MM. Piriformis muscle syndrome: an unusual adverse effect of atorvastatin. J Clin Rheumatol. 2013;19(3):156–7. 10.1097/RHU.0b013e318289ddf5. PMID: 23519180. 23519180 10.1097/RHU.0b013e318289ddf5

[65] Koda M, Mannoji C, Watanabe H, Nakajima A, Yamada T, Rokkaku T, et al. Sciatica caused by pyomyositis of the piriformis muscle. Neurol India. 2013;61(6):668–9. 10.4103/0028-3886.125291. PMID: 24441345. 24441345 10.4103/0028-3886.125291

[66] Polesello GC, Queiroz MC, Linhares JPT, Amaral DT, Ono NK. Anatomical variation of piriformis muscle as a cause of deep gluteal pain: diagnosis using MR neurography and treatment. Rev Bras Ortop. 2013;48(1):114–7. PMID: 31304122; PMCID: PMC6565897. 31304122 10.1016/j.rboe.2012.09.001 PMC6565897

[67] Sivrioglu AK, Ozyurek S, Mutlu H, Sonmez G. Piriformis syndrome occurring after pregnancy. BMJ Case Rep. 2013;2013. 10.1136/bcr-2013-008946. PMID: 23536625; PMCID: PMC3618849. bcr2013008946. 10.1136/bcr-2013-008946 PMC3618849 23536625

[68] Arooj S, Azeemuddin M. Piriformis syndrome–a rare cause of extraspinal sciatica. J Pak Med Assoc. 2014;64(8):949–51. PMID: 25252525. 25252525

[69] Menu P, Fouasson-Chaillou A, Dubois C, Dauty M. Piriformis syndrome diagnosis: on two professional cyclists. Ann Phys Rehabil Med. 2014;57(4):268–74. 10.1016/j.rehab.2014.02.006. Epub 2014 Mar 14. PMID: 24731941. 24731941 10.1016/j.rehab.2014.02.006

[70] Ortiz Sánchez VE, Charco Roca LM, Soria Quiles A, Zafrilla Disla E, Hernandez Mira F. Síndrome piramidal y variaciones anatómicas como causa de dolor ciático insidioso [Pyrimidal syndrome and anatomical variations as a cause of insidious sciatic pain]. Rev Esp Anestesiol Reanim. 2014;61(9):521-4. Spanish. 10.1016/j.redar.2014.02.010. Epub 2014 Apr 4. PMID: 24704094. 24704094 10.1016/j.redar.2014.02.010

[71] Ozisik PA, Toru M, Denk CC, Taskiran OO, Gundogmus B. CT-guided piriformis muscle injection for the treatment of piriformis syndrome. Turk Neurosurg. 2014;24(4):471-7. 10.5137/1019-5149.JTN.8038-13.1. PMID: 25050669. 25050669 10.5137/1019-5149.JTN.8038-13.1

[72] Parlak A, Aytekin A, Develi S, Ekinci S. Piriformis syndrome: a case with non-discogenic sciatalgia. Turk Neurosurg. 2014;24(1):117-9. 10.5137/1019-5149.JTN.7904-13.0. PMID: 24535806. 24535806 10.5137/1019-5149.JTN.7904-13.0

[73] Drampalos E, Sadiq M, Thompson T, Lomax A, Paul A. Intrapiriformis lipoma: an unusual cause of piriformis syndrome. Eur Spine J. 2015;24(Suppl 4):S551–4. 10.1007/s00586-014-3695-y. Epub 2014 Nov 26. PMID: 25424688. 25424688 10.1007/s00586-014-3695-y

[74] Haghnegahdar A, Sedighi M, Motalebi H. Piriformis muscle syndrome: a recurrent case after surgical release. J Surg Case Rep. 2015;2015(8):rjv105. 10.1093/jscr/rjv105. PMID: 26286539; PMCID: PMC4539510. 26286539 10.1093/jscr/rjv105 PMC4539510

[75] Moon HB, Nam KY, Kwon BS, Park JW, Ryu GH, Lee HJ, et al. Leg weakness caused by bilateral piriformis syndrome: a case report. Ann Rehabil Med. 2015;39(6):1042–6. 10.5535/arm.2015.39.6.1042. (Epub 2015 Dec 29. PMID: 26798622; PMCID: PMC4720759). 26798622 10.5535/arm.2015.39.6.1042 PMC4720759

[76] Santamato A, Micello MF, Valeno G, Beatrice R, Cinone N, Baricich A, Picelli A, Panza F, Logroscino G, Fiore P, Ranieri M. Ultrasound-Guided injection of botulinum toxin type A for piriformis muscle syndrome: A case report and review of the literature. Toxins (Basel). 2015;7(8):3045–56. 10.3390/toxins7083045. PMID: 26266421; PMCID: PMC4549739. 26266421 10.3390/toxins7083045 PMC4549739

[77] Yang HE, Park JH, Kim S. Usefulness of magnetic resonance neurography for diagnosis of piriformis muscle syndrome and verification of the effect after botulinum toxin type a injection: two cases. Med (Baltim). 2015;94(38):e1504. PMID: 26402805; PMCID: PMC4635745. 10.1097/MD.0000000000001504 PMC4635745 26402805

[78] Yıldırım P, Guler T, Misirlioglu TO, Ozer T, Gunduz OH. A case of drop foot due to piriformis syndrome. Acta Neurol Belg. 2015;115(4):847–9. 10.1007/s13760-015-0443-y. Epub 2015 Feb 14. PMID: 25676003. 25676003 10.1007/s13760-015-0443-y

[79] Zeren B, Canbek U, Oztekin HH, İmerci A, Akgün U. Bilateral piriformis syndrome in two elite soccer players: report of two cases. Orthop Traumatol Surg Res. 2015;101(8):987–90. Epub 2015 Oct 27. PMID: 26522381. 26522381 10.1016/j.otsr.2015.07.022

[80] Kraus E, Tenforde AS, Beaulieu CF, Ratliff J, Fredericson M. Piriformis syndrome with variant sciatic nerve anatomy: a case report. PM R. 2016;8(2):176–9. 10.1016/j.pmrj.2015.09.005. Epub 2015 Sep 14. PMID: 26377629. 26377629 10.1016/j.pmrj.2015.09.005

[81] Vas L, Pai R, Pawar KS, Pattnaik M. Piriformis syndrome: is it only piriformis?? Pain Med. 2016;17(9):1775–9. 10.1093/pm/pnw037. Epub 2016 Mar 19. PMID: 26995801. 26995801 10.1093/pm/pnw037

[82] Han SK, Kim YS, Kim TH, Kang SH. Surgical treatment of piriformis syndrome. Clin Orthop Surg. 2017;9(2):136–44. 10.4055/cios.2017.9.2.136. Epub 2017 May 8. PMID: 28567214; PMCID: PMC5435650. 28567214 10.4055/cios.2017.9.2.136 PMC5435650

[83] Phadke PS, Gandhi AR, More SA, Joshirao RP. Salmonella pyomyositis with concurrent sacroiliac osteomyelitis presenting as piriformis syndrome: A rare case. J Postgrad Med. 2017 Jan-Mar;63(1):44–6. 10.4103/0022-3859.192799. PMID: 27779154; PMCID: PMC5394818. 27779154 10.4103/0022-3859.192799 PMC5394818

[84] Wada K, Goto T, Takasago T, Hamada D, Sairyo K. Piriformis muscle syndrome with assessment of sciatic nerve using diffusion tensor imaging and tractography: a case report. Skeletal Radiol. 2017;46(10):1399–404. 10.1007/s00256-017-2690-x. Epub 2017 Jun 14. PMID: 28616638. 28616638 10.1007/s00256-017-2690-x

[85] Fusco P, Di Carlo S, Scimia P, Degan G, Petrucci E, Marinangeli F. Ultrasound-guided dry needling treatment of myofascial trigger points for piriformis syndrome management: a case series. J Chiropr Med. 2018;17(3):198–200. 10.1016/j.jcm.2018.04.002. Epub 2018 Aug 26. PMID: 30228811; PMCID: PMC6141415. 30228811 10.1016/j.jcm.2018.04.002 PMC6141415

[86] Ripellino P, Cianfoni A, Izzo MGA, Gobbi C. Relapsing piriformis syndrome treated with botulinum toxin injections. BMJ Case Rep. 2019;12(8):e230981. 10.1136/bcr-2019-230981. PMID: 31401586; PMCID: PMC6700580. 31401586 10.1136/bcr-2019-230981 PMC6700580

[87] Aquino-Jose VM, Blinder V, Johnson J, Havryliuk T. Ultrasound-guided trigger point injection for piriformis syndrome in the emergency department. J Am Coll Emerg Physicians Open. 2020;1(5):876–9. PMID: 33145535; PMCID: PMC7593435. 33145535 10.1002/emp2.12153 PMC7593435

[88] Fahmi A, Rahmadhan MA, Aprianto DR, Subianto H, Turchan A. Complete resolution of recurrent piriformis syndrome after piriformis resection with 3 years’ follow up: A case report. Int J Surg Case Rep. 2020;77:576–9. Epub 2020 Nov 20. PMID: 33395849; PMCID: PMC7708764. 33395849 10.1016/j.ijscr.2020.11.099 PMC7708764

[89] Hogan E, Vora D, Sherman JH. A minimally invasive surgical approach for the treatment of piriformis syndrome: a case series. Chin Neurosurg J. 2020;6:8. 10.1186/s41016-020-00189-y. PMID: 32922937; PMCID: PMC7398220. 32922937 10.1186/s41016-020-00189-y PMC7398220

[90] Koh E, Webster D, Boyle J. Case report and review of the potential role of the type A piriformis muscle in dynamic sciatic nerve entrapment variant of piriformis syndrome. Surg Radiol Anat. 2020;42(10):1237–42. 10.1007/s00276-020-02440-8. Epub 2020 Feb 28. PMID: 32112284. 32112284 10.1007/s00276-020-02440-8

[91] Leong MK, Huang P. Piriformis syndrome as the only initial manifestation of septic sacroiliac osteomyelitis. Clin Med (Lond). 2020;20(3):e18–9. 10.7861/clinmed.2020-0035. PMID: 32414734; PMCID: PMC7354056. 32414734 10.7861/clinmed.2020-0035 PMC7354056

[92] Ou Yang Y, Lo DYK, Ke Y, Lee DYH. Piriformis pyomyositis presenting as migratory hip to knee pain: a case report. JBJS Case Connect. 2020;10(4):e20.00251. 10.2106/JBJS.CC.20.00251. PMID: 33512936. 33512936 10.2106/JBJS.CC.20.00251

[93] Akbaş İ, Kocak AO, Ünlü A, Doğruyol S, Akgol Gur ST. A rare cause for sciatalgia: piriformis syndrome. Journal of Emergency Medicine Case Reports. 2021;12(3):82–4.94.

[94] Kale A, Basol G, Topcu AC, Gundogdu EC, Usta T, Demirhan R. Intrapelvic nerve entrapment syndrome caused by a variation of the intrapelvic piriformis muscle and abnormal varicose vessels: a case report. Int Neurourol J. 2021;25(2):177–80. 10.5213/inj.2040232.116. (Epub 2021 Jan 19. PMID: 33504131; PMCID: PMC8255827). 33504131 10.5213/inj.2040232.116 PMC8255827

[95] Lodin J, Brušáková Š, Kachlík D, Sameš M, Humhej I. Acute piriformis syndrome mimicking cauda equina syndrome: illustrative case. Journal of Neurosurgery: Case Lessons. 2021;2(17):CASE21252. 10.3171/CASE21252. PMID: 36060900; PMCID: PMC9435562. 36060900 10.3171/CASE21252 PMC9435562

[96] Salehi M, Ghiasvand F, Feizabadi MM, Zarei M, Yazdi NA, Alijani N, Qaempanah M, Seyedpour S. The piriformis abscess: a case-based review. Iran J Microbiol. 2021;13(2):252–6. 10.18502/ijm.v13i2.5988. PMID: 34540162; PMCID: PMC8408021. 34540162 10.18502/ijm.v13i2.5988 PMC8408021

[97] Shanmuga Jayanthan S, Rajkumar SS, Kumar VS, Shalini M. Pyomyositis of the piriformis muscle-a case of piriformis syndrome. Indian J Radiol Imaging. 2021;31(4):1023–6. 10.1055/s-0041-1739183. PMID: 35136521; PMCID: PMC8817790. 35136521 10.1055/s-0041-1739183 PMC8817790

[98] Chua E, Shah D. Hydroxyapatite crystal deposition disease around the hip: a rare cause of piriformis syndrome and ischiofemoral impingement. BJR Case Rep. 2022;7(6):20210075. 10.1259/bjrcr.20210075. PMID: 35300243; PMCID: PMC8906143. 35300243 10.1259/bjrcr.20210075 PMC8906143

[99] Güleç GG, Kurt Oktay KN, Aktaş İ, Yılmaz B. Visualizing anatomic variants of the sciatic nerve using diagnostic ultrasound during piriformis muscle injection: an example of 4 cases. J Chiropr Med. 2022;21(3):213–9. 10.1016/j.jcm.2022.02.017. PMID: 36118109; PMCID: PMC9479178. 36118109 10.1016/j.jcm.2022.02.017 PMC9479178

[100] Kaga M, Ueda T. Effectiveness of Hydro-Dissection of the piriformis muscle plus Low-Dose local anesthetic injection for piriformis syndrome: A report of 2 cases. Am J Case Rep. 2022;23:e935346. 10.12659/AJCR.935346. PMID: 35124689; PMCID: PMC8829885. 35124689 10.12659/AJCR.935346 PMC8829885

[101] Kwon SY, Jun EH, Park SJ, Kim Y. Botulinum toxin injection strategy of intractable and relapsed piriformis syndrome: a case report. Medicine. 2022;101(42):e30950. 10.1097/MD.0000000000030950. PMID: 36281083; PMCID: PMC9592348. 36281083 10.1097/MD.0000000000030950 PMC9592348

[102] Ward TRW, Garala K, Dos Remedios I, Lim J. Piriformis syndrome as a result of intramuscular haematoma mimicking cauda equina effectively treated with piriformis tendon release. BMJ Case Rep. 2022;15(3):e247988. 10.1136/bcr-2021-247988. PMID: 35236695; PMCID: PMC8895925. 35236695 10.1136/bcr-2021-247988 PMC8895925

[103] Gebregiorigis BT, Amha LG. Piriformis syndrome secondary to accessory belly of the piriformis muscle: a rare case report with MRI diagnosis. Radiol Case Rep. 2024;19(4):1503–5. 10.1016/j.radcr.2024.01.009. PMID: 38283737; PMCID: PMC10810736. 38283737 10.1016/j.radcr.2024.01.009 PMC10810736

[104] Qiu X, Luo X, Wu R. Atypical lipoma of the right piriformis muscle: a case report and review of the literature. J Med Case Rep. 2024;18(1):189. 10.1186/s13256-024-04507-1. PMCID: PMC10981807. 38555435 10.1186/s13256-024-04507-1 PMC10981807

[105] Park JW, Lee YK, Lee YJ, Shin S, Kang Y, Koo KH. Deep gluteal syndrome as a cause of posterior hip pain and sciatica-like pain. Bone Joint J. 2020;102-B(5):556–567. 10.1302/0301-620X.102B5.BJJ-2019-1212.R1. PMID: 32349600. 32349600 10.1302/0301-620X.102B5.BJJ-2019-1212.R1

[106] Hopayian K, Mirzaei M, Shamsi M, Arab-Zozani M. A systematic review of conservative and surgical treatments for deep gluteal syndrome. J Bodyw Mov Ther. 2023;36:244–50. 10.1016/j.jbmt.2022.12.003. Epub 2022 Dec 20. PMID: 37949567. 37949567 10.1016/j.jbmt.2022.12.003

[107] Othman IK, Mohamad N, Sidek S, Bhaskar RN, Kuan CS. Risk factors associated with piriformis syndrome: A systematic review. SEHS. 2020;14(3):215–33. 107. Available from:https://li01.tci-thaijo.org/index.php/sehs/article/view/241445. Cited 23 May 2024.

[108] Shah SS, Consuegra JM, Subhawong TK, Urakov TM, Manzano GR. Epidemiology and etiology of secondary piriformis syndrome: a single-institution retrospective study. J Clin Neurosci. 2019;59:209–12. 10.1016/j.jocn.2018.10.069. PMID: 30528358. 30528358 10.1016/j.jocn.2018.10.069

[109] Singh U, Meena R, Singh CA, Singh AKj S, Am, Langshong R. Prevalence of piriformis syndrome among the cases of low back/buttock pain with sciatica: a prospective study. J Med Soc. 2013;27(2):94.

[110] Nakano KK. Nerve entrapment syndromes. Curr Opin Rheumatol. 1997;9(2):165–73. 10.1097/00002281-199703000-00015. PMID: 9135923. 9135923 10.1097/00002281-199703000-00015

[111] Justice PE, Katirji B, Preston DC, Grossman GE. Piriformis syndrome surgery causing severe sciatic nerve injury. J Clin Neuromuscul Dis. 2012;14(1):45–7. 10.1097/CND.0b013e318259617c. PMID: 22922582. 22922582 10.1097/CND.0b013e318259617c

[112] Kay J, de Sa D, Morrison L, Fejtek E, Simunovic N, Martin HD, Ayeni OR. Surgical management of deep gluteal syndrome causing sciatic nerve entrapment: A systematic review. Arthroscopy. 2017;33(12):2263–e22781. 10.1016/j.arthro.2017.06.041. Epub 2017 Aug 31. PMID: 28866346. 28866346 10.1016/j.arthro.2017.06.041

[113] Vij N, Kiernan H, Bisht R, Singleton I, Cornett EM, Kaye AD, et al. Surgical and non-surgical treatment options for piriformis syndrome: a literature review. Anesth Pain Med. 2021;11(1):e112825. 10.5812/aapm.112825. (PMID: 34221947; PMCID: PMC8241586). 34221947 10.5812/aapm.112825 PMC8241586

[114] Mlinarić A, Horvat M, Šupak Smolčić V. Dealing with the positive publication bias: why you should really publish your negative results. Biochem Med (Zagreb). 2017;27(3):030201. 10.11613/BM.2017.030201. PMID: 29180912; PMCID: PMC5696751. 29180912 10.11613/BM.2017.030201 PMC5696751

[115] Bauters M, Devos P, Belzile EL, Putman S, Migaud H, Dartus J. Bibliometric evaluation of negative publications from orthopedics and traumatology from the ten most influential journals of 2009–2010 and 2019–2020: a comparative study with the orthopedics & traumatology: surgery & research journal, using the same analysis of submitted and accepted articles. Orthop Traumatol Surg Res. 2023;109(8):103703. 10.1016/j.otsr.2023.103703. Epub 2023 Oct 10. PMID: 37827451. 37827451 10.1016/j.otsr.2023.103703

